# A New Anatomic and Staging-Oriented Classification of Radical Hysterectomy

**DOI:** 10.3390/cancers13133326

**Published:** 2021-07-02

**Authors:** Mustafa Zelal Muallem

**Affiliations:** Department of Gynecology with Center for Oncological Surgery, Charité—Universitätsmedizin Berlin, Corporate Member of Freie Universität Berlin, Humboldt-Universität zu Berlin, and Berlin Institute of Health, Virchow Campus Clinic, Charité Medical University, 13353 Berlin, Germany; Mustafa-zelal.muallem@charite.de; Tel.: +49-30-450-664373; Fax: +49-30-450-564900

**Keywords:** classification of radical hysterectomy, cervical cancer, nerve-sparing radical hysterectomy, parametrium, paracolpium, tailoring the radicality, modulation of surgery

## Abstract

**Simple Summary:**

The main deficits of the available classifications of radical hysterectomy are the facts that they are based only on the lateral extension of resection, do not depend on the precise anatomy of parametrium and paracolpium and do not correlate with the tumour stage, size or infiltration in the vagina. This new suggested classification depends on the 3-dimentional concept of parametrium and paracolpium and the comprehensive description of the anatomy of parametrium, paracolpium and the pelvic autonomic nerve system. Each type in this classification tailored to the tumour stage according to FIGO- classification from 2018, taking into account the tumour size, localization and infiltration in the vaginal vault, which may make it the most suitable tool for planning and tailoring the surgery of radical hysterectomy.

**Abstract:**

The current understanding of radical hysterectomy more is centered on the uterus and little is being discussed about the resection of the vaginal cuff and the paracolpium as an essential part of this procedure. This is because that the current classifications of radical hysterectomy are based only on the lateral extent of resection. This way is easier to be understood but does not reflect the anatomical and surgical conception of radical hysterectomy and the three-dimensional ways of tumour spreading, neither meet the need of adjusting the radicality according to the different stages of FIGO classification, which depends—at least in the early stages—on the tumour volume and the infiltration in the vagina (but not on the directly spread in the parametrium). The new classification presented in this paper does not base anymore on the lateral extent of resection only but too on the depth of resection in the small pelvic and the extent of the resected vaginal vault without or with its three-dimensional paracolpium. This classification takes into account the tumour size, stage, localization and infiltration in the vaginal vault and may offer the optimal tool to adjust and tailor the surgery according to these important variables.

## 1. Introduction

Even when the radical hysterectomy has a long tradition as the standard therapy for early cervical cancer, there are a lot of unmet needs regarding the optimal technique and the most effective and practical way of defining and tailoring the radicality according to the tumour size and infiltration in the vaginal vault. The most popular classifications of radical hysterectomy are based on the lateral extent of resection (Piver [1], Querleu–Morrow [2]). This way is easier to be understood but does not reflect the anatomical and surgical conception of radical hysterectomy and the three-dimensional ways of tumour spreading, neither meet the need of adjusting the radicality according to the different stages of FIGO classification [3], which depends—at least in the early stages—on the tumour volume and the infiltration in the vagina (but not on the directly spread in the parametrium).

**Figure 1:**
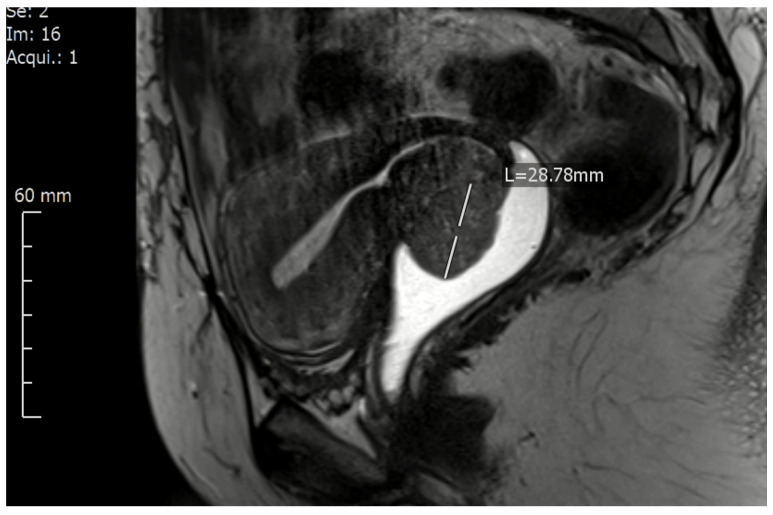
A magnetic resonance imaging of cervical cancer location on the ventral lip of the cervix (FIGO IB2) presented using a contrast agent in the vagina.

Therefore, it is essential in our opinion to find a more suitable classification for radical hysterectomy, taking into account both situations affecting the radicality and challenging the tailoring of surgery in early-stage (resectable) cervical cancer:

(1) Tumours with a big volume but without macroscopic infiltration in the vaginal vault or obvious infiltration in parametrium/paracolpium (FIGO Ib2, Ib3): these tumours demand a resection of a longer vaginal vault to be able to close the vaginal cuff beneath (caudal) from the tumour to avoid any spelling of tumour cells and any contamination of the abdominal cavity. In these cases and because of the curved and anteflexed shape of the upper vagina, tumors with ventral localization demand the resection of longer vagina cuff comparing with tumors from the same size and stage but with dorsal localization (Figure 1).
(2) Tumours infiltrating the vaginal wall with no obvious infiltration in parametrium/paracolpium (FIGOIIA1, IIA2): these tumours demand the resection of a longer vaginal vault with paracolpium (the blood supply and drain and the lymph drain of the upper 1/3 to ½ of the vagina) to be able to confirm or deny any tumour spread along with the vaginal vessels/lymph ways.

In both good operable situations, it is possible—and we have to say mandatory—to spare the pelvic autonomic nervous system to reduce the postoperative complications to the minimum [4,5,6].

The second problem in the popular classifications of radical hysterectomy is the arbitrary definition (from a surgical, anatomical and oncological point of view) of type B (Querleu–Morrow) or class II (Piver-Rutledge) radical hysterectomy as the resection of parametrium at midway/halfway, which does not correlate with tumour spread in parametrium (direct -continuous- tumour infiltration or affected lymph nodes in parametrium) and neglects the lymph nodes and ways lying at the distal part of the lateral parametrium. The continuous parametrial invasion occurs rarely, and the tumour spreading in the adjacent parametrium takes place mainly by tumour cell emboli and lymph node involvement [7]. The assessment of microscopic tumour spread in parametrium/paracolpium demands the resection and the microscopic examination of these tissues. Benedetti-Panici et al. showed in 2000 that the subclinical parametrial spreading occurred in approximately 30–60% of the apparent early stages of cervical cancer (Stage IB-IIA) [8]. This invasion was mainly a lymphovascular space infiltration and lymph node metastasis. Contiguous invasion of the parametria was less frequent. For these reasons, it is difficult to accept the resection of lateral parametrium at halfway as a sufficient tool to assess the microscopic infiltration in parametrium. Advocating this type of resection, because it can reduce the risk of ureteral injuries even when it is not radical enough to assess the microscopic parametrial invasion, is really difficult. Minimizing the risk of injuring the ureter, pelvic autonomic nerve system or the bladder and/or the ureter blood supply has to still one of the most important aspects of radical hysterectomy, but it has to depend on the precise understanding of anatomy in this area presented from Fujii et al. [9] and Muallem et al. [10]. This work aims to suggest a new anatomic and staging-oriented classification of radical hysterectomy taking into account the increased need of tailoring the surgery according to the tumor size, localization and infiltration in the vagina.

## 2. Methods

### 2.1. In-Depth Concept Explanation of the New Classification of Radical Hysterectomy

This new classification of radical hysterectomy depends on the Cibula 3-dimentional concept of parametrium [11], Muallem 3-dimentional concept of paracolpium and his comprehensive description of the anatomy of parametrium, paracolpium and the pelvic autonomic nerve system [10], and the international anatomic nomenclature (Terminologia Anatomica) [12,13].

This classification is no longer based only on the lateral extent of resection but also on the depth of resection in the small pelvic and the extent of the resected vaginal vault without or with its three-dimensional paracolpium. The resected length of the vaginal vault has to be adjusted to the tumour size (closing the vaginal vault beneath the cervical tumour to prevent any contamination of the abdominal cavity with spelling tumour cells during the resection) or to the tumour infiltration in the vagina (resecting enough vaginal margins 1–2 cm beneath the tumour infiltration in the vagina and resecting the 3-dimentional paracolpium for microscopic assessment of infiltration). This is the most crucial point by adjusting and tailoring the radicality during surgery for cervical cancers according to the tumour volume and spread. We think that the resection of all length of 3-dimentional parametrium, which it is nothing else than the lymph nodes, lymph vessels and the blood supply and drain of uterus/cervix, is essential to be performed in every FIGO-stage (up IA2) demanding a lymph node staging (Sentinel and/or lymph node dissection). Here, it is worth mentioning the contribution of Girardi [14] and Benedetti- Panici [8], who have lucidly shown that 78% to 96% of the parametrium contains lymph nodes. Pallavi and Ungar [15] and Querleu et al. [16] have further elucidated that the border between parametrial dissection and lymphadenectomy is not rigid and could be positioned in different planes while the same connective tissue is being extirpated.

This classification takes too into account the location of the cervical lesion in the cervix, which plays an important role in the surgical decision about the resected length of the vaginal vault during the radical hysterectomy. The classification distinguishes, therefore, between tumours locating on the ventral (anterior) and tumours locating on the dorsal (posterior) cervical lips in FIGO IB-stage. This is because of the fact that the resection of a longer vaginal wall ventrally, which is, of course, the case with tumours locating at the ventral cervical lip (Figure 1), is anatomically more challenging and demands at least the preparation and isolation of the proximal aspects of paracolpium to be able to resect a longer vaginal vault without injuring the inferior hypogastric plexus and the vaginal blood supply.

The radical hysterectomy has to be performed nerve sparingly in every procedure as long as there is no direct (contiguous) infiltration in the paracolpium and/or the tendinous arch of the pelvic fascia (endopelvic fascia).

### 2.2. The Three-Dimensional Anatomic Template of Parametrium and Paracolpium for the New Classification of Radical Hysterectomy

**Figure 2:**
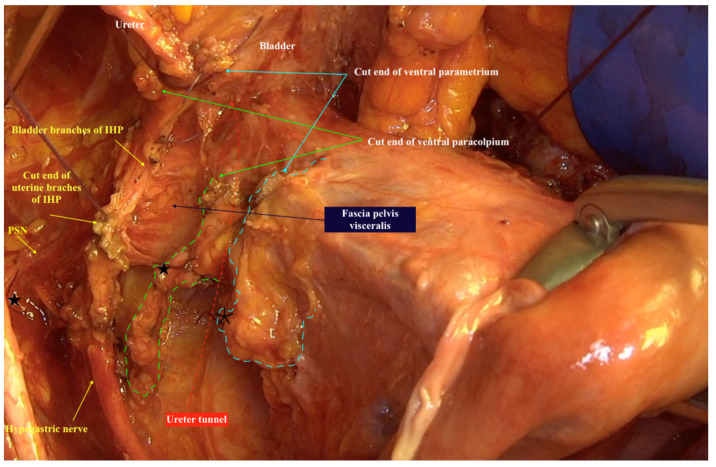
The resected three-dimensional parametrium above the ureter/ureter tunnel and the resected three-dimensional paracolpium beneath the ureter/ureter tunnel in type IV Muallem classification of radical hysterectomy. The spared left hypogastric nerve, left inferior hypogastric plexus and their branches to the bladder are even good to be seen lateral from the specimen. Parametrium marked with a dotted blue line, paracolpium marked with a dotted green line, ureter tunnel marked with a dotted red line. ★ cut end of vaginal vessels (wrongly known as deep uterine vein), * cut end of uterine vessels, IHP: inferior hypogastric plexus, PSN: pelvic splanchnic nerves.

The three-dimensional anatomic template for the resection of parametrium and paracolpium depends on the precise anatomy of parametrium and paracolpium published before [9,10] and bases on the central position of the cervix and upper vaginal in the concept of radical hysterectomy and on the ureter as a stable anatomic landmark, which splits these tissues (Paracervix) to parametrium (above = cranial and craniomedial from the ureter) and paracolpium (beneath = caudal and caudolateral from the ureter, Figure 2). 

**Figure 3:**
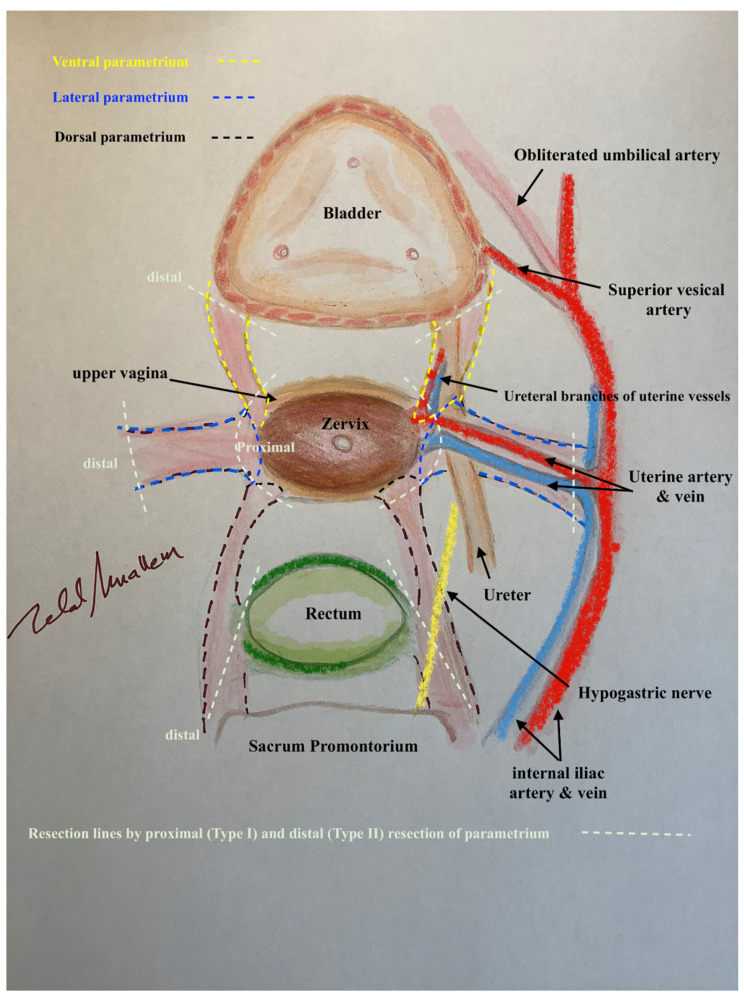
The three-dimentional parametrium in a cross-section of midpelvis with the explanation of distal and proximal aspects of ventral, lateral and dorsal parametrium and the resulted type I and type II radical hysterectomy.

**Figure 4:**
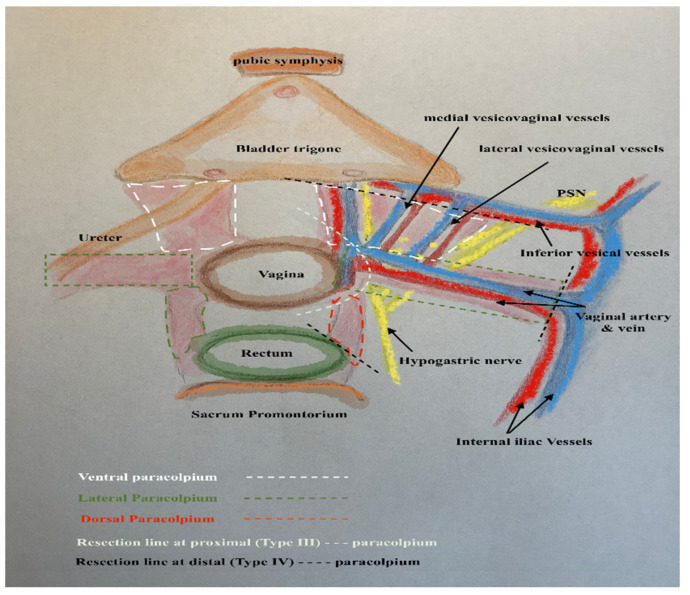
The three-dimentional paracolpium in a cross-section direct above the pelvic floor with the explanation of distal and proximal aspects of ventral, lateral and dorsal paracolpium and the resulted type III and type IV radical hysterectomy.

In this way, every part of the three-dimentional parametrium and paracolpium has a proximal aspect (at the cervix in the case of parametrium and at the vaginal wall in the case of paracolpium) and a distal aspect (at the bladder trigone in case of ventral parametrium and ventral paracolpium, at the internal iliac vessels in the case of lateral parametrium and lateral paracolpium, and at the rectum sidewall and sacrum in case of dorsal parametrium and dorsal paracolpium). Figure 3 shows the three-dimensional parametrium in a cross-section of the midpelvis, and Figure 4 shows the three-dimensional paracolpium in a cross-section directly above the pelvic floor.

The dorsal parametrium in this classification is the sacrouterine ligament, and the dorsal paracolpium is the sacrovaginal ligament which has been previously described as the deep uterosacral ligament by Ramanah et al. [17] and as the vaginorectal ligament by various other authors [18]. Laterally, we recognize the lateral parametrium above the ureter, containing the uterine artery and vein, and the lateral paracolpium beneath the ureter, containing the vaginal artery and vein (wrongly known as deep uterine vein).

**Figure 5:**
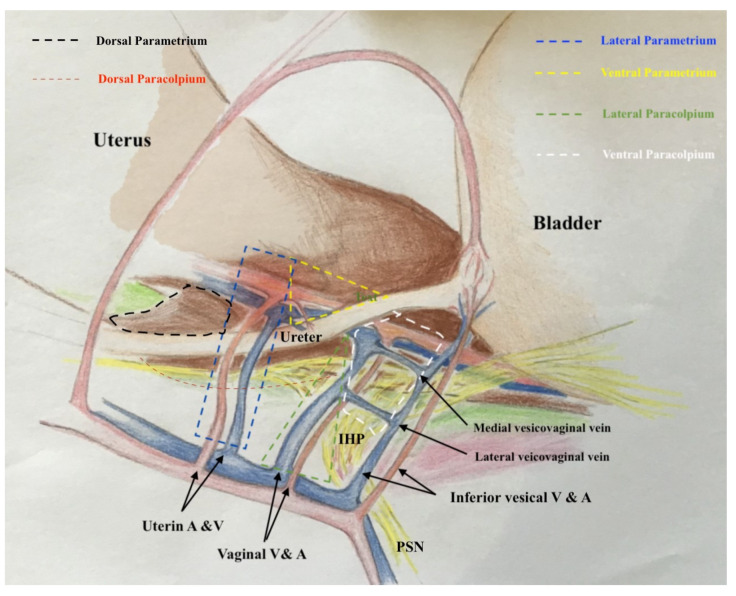
The graphic explanation of detailed anatomy of parametrium and paracolpium; dotted black line: dorsal parametrium, dotted blue line: lateral parametrium, dotted yellow line: ventral parametrium, dotted red line: dorsal paracolpium, dotted green line: lateral paracolpium and dotted white line: ventral paracolpium.

The ventral parametrium (cranial and medial portions from the distal ureter) is the vesicouterine ligament, and the ventral paracolpium (lateral and caudal portions from the distal ureter) is the vesicovaginal ligament which contains the venal anastomoses between the vaginal vein and the inferior vesical vein (the vesical venous plexus) consisting from the lateral and medial vesicovaginal veins (known too as middle and inferior vescical vein [9])—Figure 5.

The detailed description of parametrium and paracolpium is shown in Table 1.

## 3. Results (The New Classification and Its Clinical Impacts)

The new classification of radical hysterectomy describes four types of radical hysterectomy according to the clinical and surgical demands. Each type in this classification is tailored to the tumour stage according to the International Federation of Gynecology and Obstetrics (FIGO)- classification from 2018, taking into account the tumour size, localization and infiltration in the vaginal vault and depends on the precise three-dimensional anatomy of parametrium and paracolpium and their close anatomic relationships to the pelvic autonomic nerve system. The pelvic exentration or the extended resection of pelvic organs (Class V in Piver-classification or Type D in Querleu-Morrow classification) is no more a part of this classification because this kind of radical resection is rarely indicated for primary cases and is not compatible with the concept of radical hysterectomy.

### 3.1. Type I (The Limited Radical Hysterectomy)

**Figure 6:**
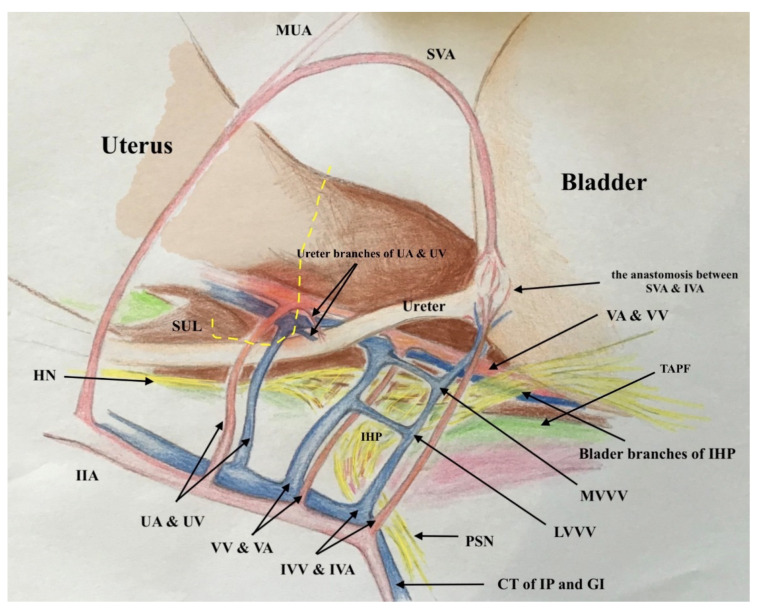
The graphic explanation of type I radical hysterectomy according to Muallem classification; the dotted yellow line shows the resection line at the proximal aspects of the ventral, lateral and dorsal parametrium.

This procedure is equivalent to the so-called extra-fascial hysterectomy (Class A in Q-M classification) aiming to remove the entire uterus with parmetrium margins (only the proximal aspects of 3-dimensional parametrium) and minimal vaginal vault. This tailored procedure is suitable for microscopic cervical cancers FIGO IA (no need for covering vaginal vault) and no lymphovascular space invasion (LVSI) (Figure 6).

### 3.2. Type II (The Typical Radical Hysterectomy)

**Figure 7:**
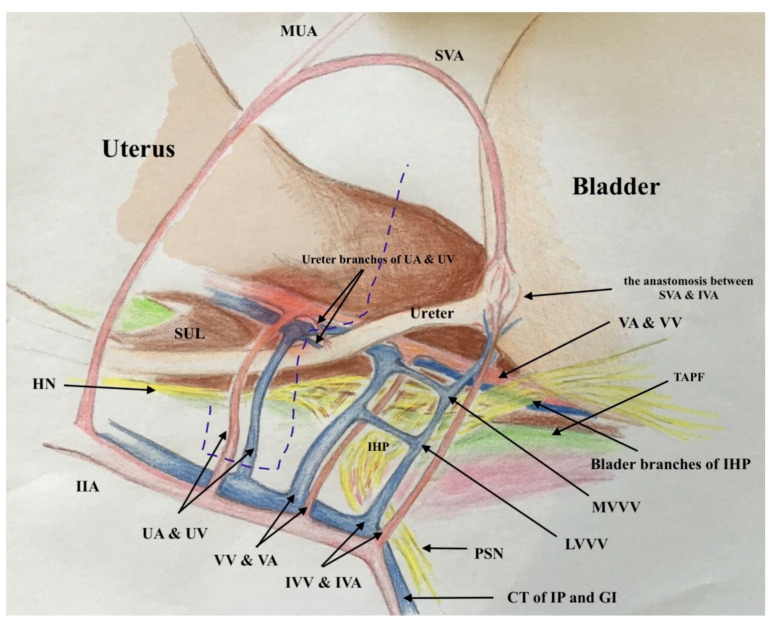
The graphic explanation of type II radical hysterectomy according to Muallem classification; the dotted blue line shows the resection line at the distal aspects of the ventral, lateral and dorsal parametrium. No needed resection of paracolpium.

This procedure is equivalent to the Wertheim-Meigs Operation and included the resection of the distal aspects of the new defined ventral (at the ureteral junction to the bladder at the vesical trigone), lateral (at the internal iliac vessels) and dorsal parametrium (at the rectum sidewall) but without paracolpium resection and with minimal (about 2 cm) vaginal vault, which will be the surgery of choice for stages IA with lymphovascular space invasion and IB1 (less than 2 cm) with dorsal localization (Figure 7).

### 3.3. Type III (The Typical Radical Hysterectomy with Extended Vaginal-Cuff-Resection)

**Figure 8:**
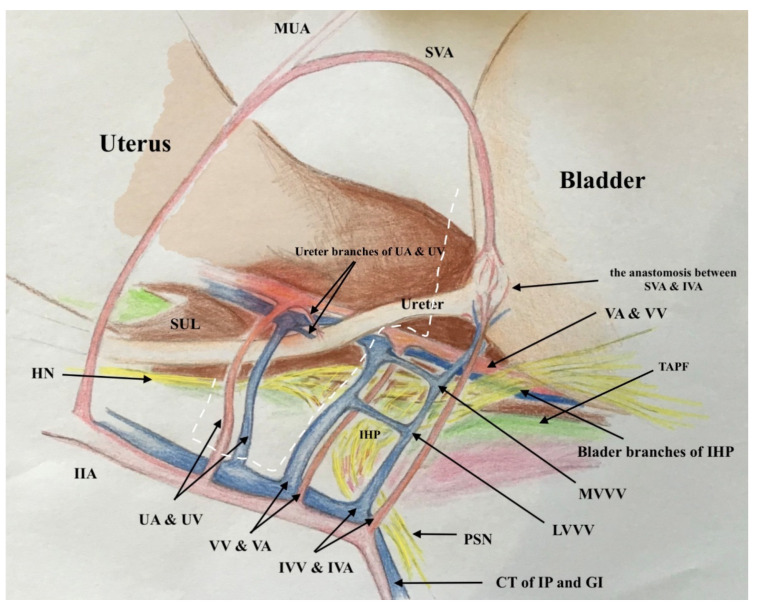
The graphic explanation of type III radical hysterectomy according to Muallem classification; the dotted white line shows the resection line at the distal aspects of ventral, lateral and dorsal parametrium and at the proximal aspects of ventral, lateral and dorsal paracolpium.

This procedure is similar to the class C1 radical hysterectomy in Q-M classification with complete resection of the upper defined ventral, lateral and dorsal parametrium but with resecting of only the proximal aspects 3-dimentional paracolpium allowing the resection of the longer vaginal vault (about 2–4 cm). This demands more preparation of the vagina and highlighting the vaginal vessels and the ventral parts of the inferior hypogastric plexus to be able to dissect them carefully, lateralizing and sparing them during the procedure. This type of radicality is the surgery of choice for stage IB1 with ventral localization, IB2 and IB3 with dorsal localization in the cervix. This new type of radical hysterectomy has the advantage of avoiding the difficult preparation and resection of the vesical venous plexus (lateral and medial vesicovaginal veins) and the vaginal vessels, which reduce the risk of injuring the inferior vesical vessels and the following ischemic injuries of the distal ureter and the bladder trigon. This type offers the opportunity to resect an adapted length of vaginal vault to cover big cervical tumours (Figure 8). It is worth mentioning that this type will not be radical enough when there is any macroscopically vaginal infiltration. This case demands the 3-dimensional resection of paracolpium (type IV).

### 3.4. Type IV (The Radical Hysterectomy with Radical Upper Colpectomy)

**Figure 9:**
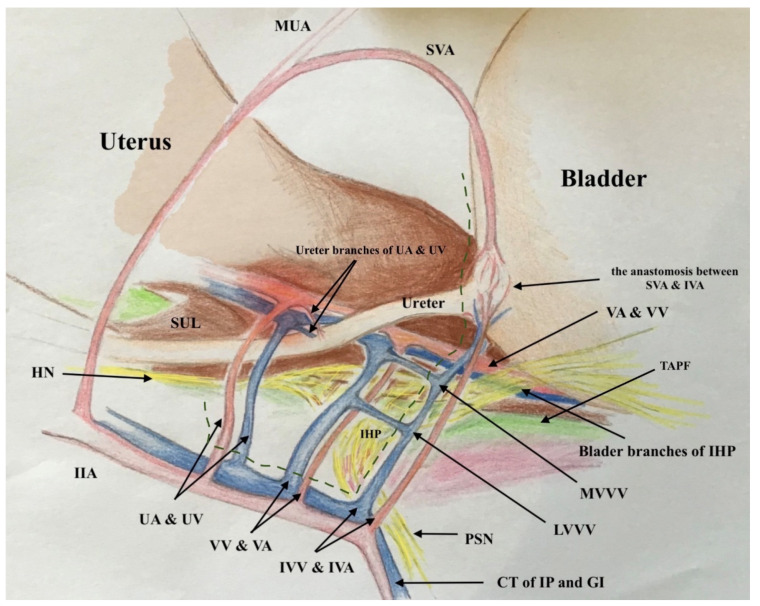
The graphic explanation of the type IV radical hysterectomy according to Muallem classification; the dotted green line shows the resection line at the distal aspects of ventral, lateral and dorsal parametrium and at the distal aspects of ventral, lateral and dorsal paracolpium.

This procedure is equivalent to the nerve-sparing Okabayashi operation (modified from Fujii) and included the radical hysterectomy with resection of the distal aspects of ventral (at the ureteral junction to the bladder at the vesical trigone), lateral (at the internal iliac vessels) and dorsal parametrium (at the rectum sidewall) with the resection of distal aspects of ventral paracolpium (at the inferior vesical vessels with resection of lateral and medial vesicovaginal vessels), lateral paracolpium (at the internal iliac vessels) and dorsal paracolpium (at the tendinous arch of the pelvic fascia) (Figure 9). This procedure has to be performed nerve sparingly as long as there is no direct (contiguous) infiltration in the paracolpium and/or the tendinous arch of the pelvic fascia (endopelvic fascia).

In the direct infiltration in paracolpium and/or in the endopelvic fascia, the ipsilateral resection of the inferior hypogastric plexus will be mandatory. This procedure is the surgery of choice for stage IB3 with ventral localization or deep stromal invasion, stage IIA and selected cases of stage IIB.

**Figure 10:**
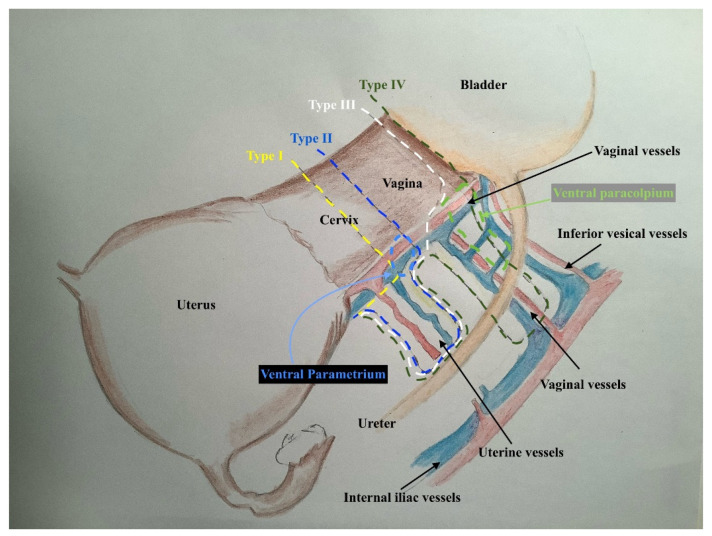
The graphic explanation of all types of a new classification of radical hysterectomy.

Figure 10 presents the differences between the 4 types of radical hysterectomy proposed in this study with different length of resected Parametrium, paracolpium and vagina. 

Table 2 explains the types of radical hysterectomy according to this new classification.

## 4. Discussion

The old classifications of radical hysterectomy depended only on the lateral extension of resection. This misinterpreted the concept of surgery to be reduced only on the resection of lateral parametrium with no or minimal resection of the ventral parametrium. The limited resection of the ventral parametrium and/or paracolpium restricted the length of the resected vaginal vault and made the surgery of tumours with big volumes (>2 cm) or with vaginal invasion pretty difficult.

The new classification is based not only on the lateral extension of resection, but also on the depth of resection in the small pelvis, taking into account the three-dimensional parametrium and paracolpium template and the comprehensive description of the anatomy of parametrium, paracolpium and the pelvic autonomic nerve system [10]. It takes into account the new FIGO- the staging of cervical cancer and tailors the surgery according to the FIGO-stage, the tumour localization on the cervix uteri, the tumour volume and the tumour spread in the vaginal cuff.

The author is aware that this new classification does not result from randomized control studies, but it depends on a clear described anatomical and surgical concept [5,6,10] and on the long experience of operating radical hysterectomy. It is worth to remind here that the available classifications of radical hysterectomy still not supported with randomized-control trials. The current practice on radical hysterectomy suffers under a variety of tailored surgical procedures apart from standard radical hysterectomy without clear evidence for the oncological safety of all these modifications. Even when we have two randomized control studies on more or less radical parametrectomy, which showed that oncological safety was not compromised by doing less radical surgery, because of the heterogeneity of the patient population and the high frequency of adjuvant radiotherapy, the true impact of surgical radicality cannot be assessed [19]. The outcomes of LACC-Trial [20] give rise to more awareness about the importance of the prevention of tumour cell contamination and avoiding the intracorporal colpotomy before closing the vaginal vault beneath the cervical tumour [21,22,23,24]. This makes it mandatory to emphasize the crucial role of resection an adapted enough length from the vagina according to the tumour size and infiltration in the vaginal cuff. The infiltration in the vaginal cuff increases the risk of microscopic tumour spread in paracolpium (the blood supply, lymph nodes and the lymph drains of the upper vagina), therefore it is essential in such cases (FIGO IIA) to resect the three-dimensional paracolpium for diagnostic and therapeutic purposes. This classification does not consider the nerve-sparing radical hysterectomy as an option but as a standard therapy of care, as the anatomical and surgical studies [5,6,10] confirmed the feasibility of nerve-sparing radical hysterectomy in all stages if there is no direct (contiguous) infiltration in the paracolpium and/or the tendinous arch of the pelvic fascia (endopelvic fascia).

This new classification may supply a very good tool for uniting the terminology and definitions of radical hysterectomy and for planning the right tailored radical surgery for cervical cancer according to the tumour size, stage, localization and infiltration in the vaginal vault. All these parameters could be evaluated with clinical examination and with or without additional magnetic resonance imaging.

## 5. Conclusions

The new suggested classification of radical hysterectomy does not depend only on the lateral extension of resection in lateral parametrium but consider the three-dimensional template of parametrial resection, the three-dimensional template of resection of paracolpium and the comprehensive description of the anatomy of parametrium, paracolpium and the pelvic autonomic nerve system. It may be a good tool for planning and tailoring the surgery according to the tumour size, stage, localization and infiltration in the vaginal vault.

## Figures and Tables

**Table 1:** Definitions of parametrium and paracolpium components and detailed description of their proximal and distal aspect as well as the cranial and caudal limits.

Components	Definition	Distal Aspect	Proximal Aspect	Cranial Limit	Caudal Limit
Ventral parametrium	Vesicouterine ligament (the uterine branches to ureter)	Ureteral junction to the bladder (at the vesical trigone)	Uterine vessels at the cervical sidewall	The peritoneum of vesicouterine fold	Ureter junction to the bladder
Lateral parametrium	Uterine vessels, uterine lymph nodes and ways.	Internal iliac vessels	Uterine vessels at the cervical sidewall	Superior vesical artery/medial umbilical ligament	Ureter tunnel/The vaginal vessels
Dorsal parametrium	Sacrouterine ligament	at the rectum sidewall	The cervical sidewall	The peritoneum of sacrouterine ligament	The pelvic nerve-vessel guiding plate (level of hypogastric nerve)
Ventral paracolpium	Vesicovaginal ligament (the anastomosis between vaginal vessels and inferior vesical vessels)	Ureteral junction to the bladder/inferior vesical vessels	Vaginal vessels at the vaginal sidewall	Ureter junction to the bladder	The bladder branches of inferior hypogastric plexus/Fascia pelvis vesceralis
Lateral paracolpium	Vaginal vessels and vaginal lymph nodes and ways	Internal iliac vessels	Vaginal vessels at the vaginal sidewall	Ureter tunnel/uterine vessels = lateral parametrium	Pelvic splanchnic nerves/pelvic floor
Dorsal paracolpium	Sacrovaginal ligament	The rectum sidewall/Sacrum	The vaginal sidewall	Dorsal parametrium = sacrouterine ligament	Fascia pelvis vesceralis

**Table 2:** Types of radical hysterectomy in Muallem classification with the clinical indication and the expected length of the resected vaginal vault.

Type of Radical Hysterectomy	I	II	III	IV
Ventral parametrium	At the proximal aspect	At the distal aspect	At the distal aspect	At the distal aspect
Lateral parametrium	At the proximal aspect	At the distal aspect	At the distal aspect	At the distal aspect
Dorsal parametrium	At the proximal aspect	At the distal aspect	At the distal aspect	At the distal aspect
Ventral paracolpium	-	-	At the proximal aspect	At the distal aspect
Lateral paracolpium	-	-	At the proximal aspect	At the distal aspect
Dorsal paracolpium	-	-	At the proximal aspect	At the distal aspect
Vaginal vault	Minimal/at the cervical boarders	1–2 cm	2–4 cm	2 cm beneath the tumour.
Indication/FIGO-Stage	Ia (no LVSI)	Ia + LVSI, Ib1 with dorsal localization	Ib1 with ventral localization, Ib2, Ib3 with dorsal localization	Ib3 with ventral localization, IIa, IIb

## Data Availability

No new data were created or analyzed in this study. Data sharing is not applicable to this article.
